# Use of antidepressant medications in relation to the incidence of breast cancer

**DOI:** 10.1038/sj.bjc.6603017

**Published:** 2006-03-07

**Authors:** D Fulton-Kehoe, M A Rossing, C Rutter, M T Mandelson, N S Weiss

**Affiliations:** 1 Department of Environmental and Occupational Health Sciences, University of Washington, School of Public Health and Community Medicine, Seattle, WA, USA; 2 Department of Epidemiology, University of Washington, School of Public Health and Community Medicine, Seattle, WA, USA; 3 Division of Public Health Sciences, Fred Hutchinson Cancer Research Center, Seattle, WA, USA; 4 Center for Health Studies, Group Health Cooperative, Seattle, WA, USA

**Keywords:** breast cancer, antidepressant agents, case-control studies

## Abstract

Although associations have been reported between antidepressant use and risk of breast cancer, the findings have been inconsistent. We conducted a population-based case–control study among women enrolled in Group Health Cooperative (GHC), a health maintenance organization in Washington State. Women with a first primary breast cancer diagnosed between 1990 and 2001 were identified (*N*=2904) and five controls were selected for each case (*N*=14396). Information on antidepressant use was ascertained through the GHC pharmacy database and on breast cancer risk factors and screening mammograms from GHC records. Prior to one year before diagnosis of breast cancer, about 20% of cases and controls had used tricyclic antidepressants (adjusted odds ratio=1.06, 95% CI 0.94–1.19) and 6% of each group had used selective serotonin reuptake inhibitors (OR=0.98, 95% CI 0.80–1.18). There also were no differences between cases and controls with regard to the number of prescriptions filled or the timing of use. Taken as a whole, the results from this and other studies to date do not indicate an altered risk of breast cancer associated with the use of antidepressants overall, by class, or for individual antidepressants.

Antidepressant use has increased steadily (Stafford *et al*, 2001; Pirraglia *et al*, 2003; Meijer *et al*, 2004) since the introduction of selective serotonin reuptake inhibitors (SSRIs) in the late 1980s, with use reported by 10–20% of women in recent studies (Moorman *et al*, 2003; Steingart *et al*, 2003; Chien *et al*, 2005), though actual use is likely to be higher due to underreporting (Boudreau *et al*, 2004).

Antidepressants may affect the risk of breast cancer through the influence of prolactin, of which elevated levels have been observed with use of SSRIs, tricyclic antidepressants, and other psychotropic drugs (Amsterdam *et al*, 1997; Cowen and Sargent, 1997; Emiliano and Fudge, 2004). Higher prolactin levels have been associated with an increased risk of postmenopausal breast cancer (Hankinson *et al*, 1999; Tworoger *et al*, 2004). However, the results of several studies of a possible association between antidepressant use and breast cancer risk have been inconsistent (see Discussion section).

To assess more completely the relation of breast cancer with antidepressant use, we conducted a large case control study using both computerized pharmacy records as well as records of reproductive history, family history of breast cancer, and other breast cancer risk factors. Breast cancer cases diagnosed during 1990–2001 were included, which allowed examination of newer SSRIs as well as tricyclic antidepressants.

## METHODS

All study subjects were enrollees of Group Health Cooperative (GHC), a health maintenance organization with over 500 000 members in western Washington State. Women with a new diagnosis of invasive or *in situ* breast cancer diagnosed between 1990 and 2001 were identified by routine linkage between the GHC enrollment file and the Seattle-Puget Sound Surveillance, Epidemology, and End-Results SEER (2005). Five controls were selected for each case matched on age, calendar year, and length of GHC membership, with the diagnosis date of the case assigned to each matched control as the reference date. To ensure more complete pharmacy records, we restricted cases and controls to women continuously enrolled at GHC for at least 4 years. We identified 2904 eligible cases age 30–79 when diagnosed with a first primary invasive or *in situ* breast cancer and 14396 eligible controls.

Since 1977, the GHC pharmacy database has included a record for all prescriptions dispensed to GHC enrollees. Each pharmacy record includes a patient identifier, the drug type and dose, date dispensed, quantity dispensed, and dosing instructions. Measures of antidepressant exposure included all prescriptions from the GHC enrollment start date up to 1 year prior to the reference date, with the exception of time since last use, which also included prescriptions filled in the year before the reference date. To provide some assurance that a prescribed drug was actually taken, we classified women as antidepressant users if they filled at least two prescriptions in a 6-month interval. We used number of prescriptions filled as a measure of duration of use, with a typical prescription filled for 1 month.

We examined antidepressant use overall, individual antidepressant medications, and four classes of antidepressants including SSRIs, tricyclics, monoamine oxidase inhibitors (MAOIs), and atypical antidepressants. The SSRI category included paroxetine, fluoxetine, sertraline, fluvoxamine, and citalopram. Tricyclic antidepressants included amitriptyline, doxepin, imipramine, desipramine, nortriptyline, protriptyline, amoxapine, trimipramine, and clomipramine. The MAOIs included phenelzine and tranylcypromine. Bupropion, mirtazapine, nefazodone, venlafaxine, maprotiline (a tetracyclic antidepressant), and trazodone were classified as ‘atypical’ antidepressants.

Information on breast cancer risk factors and mammography history were obtained from the GHC Breast Cancer Screening Program (BCSP) databases (Taplin *et al*, 1990). As part of the Breast Cancer Screening Program, self-administered questionnaires are first sent to all GHC women when they turn 40 (or enroll in GHC, whichever is later). Of eligible women, approximately 85–90% enroll in the BCSP and complete the initial questionnaire. The questionnaire includes information on clinical breast exams, previous mammograms, menopausal status, use of menopausal hormone replacement therapy, age at first birth, previous biopsies, family history of breast cancer, height, weight, education, and race. Selected questions from the BCSP questionnaire are updated at each screening visit. GHC also maintains databases of mammograms performed at GHC with dates of each mammogram, indications for examination (screening or diagnostic), type of evaluation (standard screening or diagnostic), results, and recommendations. Based on these records, we determined whether a study participant had had a screening mammogram in the 2 years prior to her diagnosis or reference date.

Unconditional logistic regression was used to compute odds ratios (as an estimate of relative risk) and 95% CI for duration and type of antidepressant use in relation to risk of breast cancer. All models included the matching factors (age at reference date, length of GHC membership, and reference year) as continuous variables. Family history of breast cancer, use and duration of HRT, body mass index, parity/age at first birth, and mammographic screening in the 2 years before reference date were determined to be potential confounders based on their associations with both exposure and disease. In addition to the matching factors, these factors were included in all multivariate models.

Our primary analyses included all women with invasive breast cancer and controls. Additional analyses examined ductal and lobular cases separately. We also conducted analyses separately of women with oestrogen receptor (ER) positive and ER negative tumours, based on routinely collected information from SEER. Polytomous regression models were used when cases were classified based on histology or ER status, which resulted in multiple case groups. To ensure more complete exposure data, some analyses were restricted to the subgroup of women enrolled in GHC for at least 10 years (over 70% of eligible subjects in this study). Cases with *in situ* disease were examined separately due to differences in the natural history (Bodian, 1993; Jensen and Page, 2003) and in some risk factors (Weiss *et al*, 1996; Trentham-Dietz *et al*, 2000; Moorman *et al*, 2003) between invasive and *in situ* disease.

## RESULTS

Of the 2904 eligible cases, 2449 had invasive breast cancer and 455 had *in situ* carcinoma. Of the cases with invasive disease, 72% were ductal, 14% lobular or mixed lobular and ductal, and 14% were other histologies. Compared to controls, women with invasive breast cancer were more likely to report a family history of breast cancer, to be nulliparous, and to be at least 30 years old at first birth (Table 1). A higher percent of cases reported ever use of HRT and longer use of HRT. Women with invasive disease were slightly more likely to be overweight or obese than controls. The percentage of subjects with a screening mammogram within 2 years of the reference date was higher for invasive cases (71%) than for controls (58%) and cases were more likely to report a previous breast biopsy than controls.

Overall, 34% of women had filled at least 1 prescription for antidepressants up to 1 year before the diagnosis or reference date. About 24% had filled at least 2 antidepressant prescriptions within 6 months and were considered antidepressant users for this study. About 20% had filled at least 2 prescriptions for tricyclic antidepressants. For SSRIs, 6% had filled at least two prescriptions within 6 months and about 6% filled at least two prescriptions for atypical antidepressants. For each antidepressant class, women filled a median of 10 prescriptions.

Among controls, women who used antidepressants were more likely to report use of HRT than nonusers and had a longer duration of use than women who had not used antidepressants (Table 2). A higher percentage of women who used antidepressants were classified as obese than those who did not use antidepressants. Antidepressant users were also more likely to have had a screening mammogram within 2 years before the reference date and had been enrolled at GHC for a longer time than nonusers. Women who were older, had a younger age at menarche, a first birth before 20 years of age, or were white were more likely to use antidepressants, although these differences were relatively small.

When only adjusted for matching factors, the odds ratio for ever use of antidepressants was slightly elevated (OR=1.11 95% CI 1.00–1.22, *P*=0.048) (Table 3). After adjusting for potential confounding variables the OR was 1.04 (95% CI=0.94–1.16). Risk did not increase with increasing number of antidepressant prescriptions filled. The odds ratio for tricyclic use was slightly elevated when adjusted only for matching factors (OR=1.12, 95% CI 1.00–1.24), but not after additional adjustment (OR=1.06, 95% CI 0.94–1.19). The risk was not elevated with a greater number of tricyclic prescriptions filled. Ever use of SSRIs also was not associated with breast cancer risk (adjusted OR=0.98, 95% CI 0.80–1.18), nor was receipt of a relatively larger number of prescriptions. Use of atypical antidepressants was not associated with an increased risk of breast cancer.

In the analyses of the three most common tricyclic medications (Table 4), there was no association with doxepin or imipramine, but the odds ratios were somewhat elevated for amitriptyline. When accounting only for the matching factors, the odds ratio for ever use of amitriptyline was 1.27 (95% CI 1.10–1.47). The odds ratio was somewhat attenuated, but still slightly elevated (OR=1.21, 95% CI 1.03–1.41) after adjusting for potential confounding factors. No elevated risks were observed for the three most common SSRIs (fluoxetine, paroxetine, or sertraline) or with trazodone, the most common atypical antidepressant.

No patterns of increased risk were seen with time since first use or time since last use of tricyclics or SSRIs (data not shown).

In analyses by ER status, no associations were seen between antidepressant use overall, number of antidepressant prescriptions, SSRI use, number of SSRI prescriptions, tricyclic use, or number of tricyclic prescriptions with either ER+ or ER− breast cancer (Table 5). With the possible exception of amitriptyline, individual antidepressants were not associated with either ER+ or ER− breast cancer. In fully adjusted models for ER+ breast cancer, the OR for ever use of amitriptyline was 1.22 (95% CI 1.02–1.45). For 2–10 prescriptions of amitriptyline the OR was 1.19 (95% CI 0.95–1.48) and for 11 or more prescriptions the OR was 1.27 (95% CI 0.96–1.66). No associations were observed with other individual antidepressant medications (data not shown).

In the analysis of cases with invasive ductal carcinoma (data not shown), the odds ratios for the highest category of antidepressant use (OR=1.31, 95% CI 1.02–1.68) and the highest category of tricyclic use (OR=1.31, 95% CI 1.00–1.88) were somewhat elevated after adjusting for potential confounders. Risk was not elevated in any of the other categories of use, with no indication of a dose–response trend. No associations were seen between risk of ductal carcinoma and use of SSRIs or atypical antidepressants. Antidepressant use was not associated with invasive lobular carcinoma.

When analyses were restricted to women enrolled in GHC for at least 10 years, risks did not differ for antidepressants overall or any class of antidepressants (data not shown). Neither any use of antidepressants overall nor use of a particular class of antidepressants was associated with an increased risk of carcinoma *in situ* (data not shown).

## DISCUSSION

With few exceptions, no increased risks were observed between antidepressant use and risk of invasive breast cancer. After adjustment for potential confounding factors no associations were seen between risk of breast cancer and antidepressants overall, SSRIs, tricyclics, or atypical antidepressants. No increased risk was observed with time since first or last use of any class of antidepressants. When individual antidepressants were examined, only amitriptyline exhibited a possible, albeit weak, association (OR=1.21, 95% CI 1.03–1.41). Risks were not elevated for any other individual medications.

Similar to our study, most prior studies have found little or no association with ever use of antidepressants (all classes combined) (Cotterchio *et al*, 2000; Wang *et al*, 2001; Moorman *et al*, 2003; Steingart *et al*, 2003; Chien *et al*, 2005), with increasing duration of use (Cotterchio *et al*, 2000; Wang *et al*, 2001; Moorman *et al*, 2003; Steingart *et al*, 2003; Chien *et al*, 2005), or with time since first or last use (Steingart *et al*, 2003; Chien *et al*, 2005).

For use of tricyclic antidepressants, no increased risks were seen in most studies for current (Chien *et al*, 2005; Gonzalez-Perez and Garcia Rodriguez, 2005) or ever use (Kelly *et al*, 1999; Cotterchio *et al*, 2000; Wang *et al*, 2001; Sharpe *et al*, 2002; Moorman *et al*, 2003; Steingart *et al*, 2003; Chien *et al*, 2005). In the present study, there was a weak positive association when adjusted only for matching factors and this was attenuated when adjusted for additional potential confounders. Longer duration of tricyclic use was associated with an increased risk of breast cancer (OR=2.1, 95% CI 0.9–5.0) in one study (Cotterchio *et al*, 2000), but not in others (Moorman *et al*, 2003; Gonzalez-Perez and Garcia Rodriguez, 2005), including the present study, although cutpoints for the highest category of use have varied. Although we found a slightly increased risk with use of amitriptyline, other studies that examined individual tricyclic medications observed no increased risks associated with use of this drug (Kelly *et al*, 1999; Cotterchio *et al*, 2000; Wang *et al*, 2001; Steingart *et al*, 2003). Sharpe *et al* (2002) reported an elevated risk of breast cancer associated with the tricyclic drugs classified as ‘genotoxic’ based on *Drosophila* wing development (van Schaik and Graf, 1991; van Schaik and Graf, 1993). In the present study, no increased risk was observed either with an increasing number of prescriptions or timing of use of these same potentially genotoxic tricyclic medications (data not shown). In fact, in contrast to the findings by Sharpe *et al*, we found a slightly elevated risk of breast cancer associated with use of nongenotoxic tricyclic drugs, which includes the drug amitriptyline.

Most studies (Kelly *et al*, 1999; Cotterchio *et al*, 2000; Wang *et al*, 2001; Moorman *et al*, 2003; Coogan *et al*, 2005; Gonzalez-Perez and Garcia Rodriguez, 2005) have not found an association between ever use of SSRIs and risk of breast cancer, but one reported a slight increased risk (OR=1.32, 95% CI 0.97–1.80) (Steingart *et al*, 2003). Although recent use of SSRIs was associated with an increased risk of breast cancer in one study (Kelly *et al*, 1999), our study and others (Chien *et al*, 2005; Gonzalez-Perez and Garcia Rodriguez, 2005) that examined timing of use did not find a similar association. Duration of SSRI use has been assessed in this and other studies (Kelly *et al*, 1999; Cotterchio *et al*, 2000; Moorman *et al*, 2003; Chien *et al*, 2005; Coogan *et al*, 2005; Gonzalez-Perez and Garcia Rodriguez, 2005). One reported a doubling of the risk (OR=2.2, 95%CI=0.8–6.3) with duration of use longer than 3 years (Moorman *et al*, 2003), but others have not observed an increased risk with increasing duration of use, although few women had used SSRIs for more than 2–3 years.

Individual SSRI medications have been associated with an increased risk of breast cancer in two previous studies (Cotterchio *et al*, 2000; Steingart *et al*, 2003), but not in others (Chien *et al*, 2005; Coogan *et al*, 2005; Gonzalez-Perez and Garcia Rodriguez, 2005; Haque *et al*, 2005) including our current study. Although one study reported an elevated odds ratio associated with use of sertraline (OR=1.45, 95% CI 0.88–2.40) (Steingart *et al*, 2003), in our study and in others (Cotterchio *et al*, 2000; Coogan *et al*, 2005) the risk was not increased. The risk associated with paroxetine use has been substantially (OR=7.2, 95% CI 0.9–58.3) (Cotterchio *et al*, 2000) and moderately (OR=1.45, 95% CI 0.88–2.40) (Steingart *et al*, 2003) elevated in two studies, but not in ours or others (Chien *et al*, 2005; Coogan *et al*, 2005; Gonzalez-Perez and Garcia Rodriguez, 2005; Haque *et al*, 2005). We did not find an association with ever use of paroxetine (OR 1.00) or with increasing number of paroxetine prescriptions filled (OR 1.15 for 2–10 prescriptions and 0.52 for at least 11 prescriptions).

Atypical antidepressants have not shown an increased risk in this or other studies (Moorman *et al*, 2003; Chien *et al*, 2005). Trazodone was a commonly used antidepressant in our study population, but the number of women reporting use of atypical antidepressants has been quite low in other studies. Some studies relying on self-report may underestimate trazodone use if other indications of antidepressant use (such as insomnia) are not assessed.

The one other study that evaluated whether the risk associated with antidepressant use varies by ER status reported associations of antidepressant use with ER− tumors and with PR− tumors. The strongest association was observed with SSRIs in ER+/PR− cases (OR=2.0, 95% CI 1.1–3.8)(Chien *et al*, 2005). We did not find an association between SSRIs, tricyclics, or antidepressant use overall with either ER+ or ER− breast cancers. In contrast to the earlier study (Chien *et al*, 2005), when we examined ER/PR combinations, risks were not elevated for ER+/PR− cases (data not shown).

Recently, one study reported that antidepressant users had a lower risk of breast cancer among women with a family history of breast cancer and a higher risk in those without a family history (Chien *et al*, 2005). Our results do not replicate this interaction (data not shown).

Much of the prior research examining antidepressants and breast cancer risk has relied on the self-reported use of antidepressants (Kelly *et al*, 1999; Cotterchio *et al*, 2000; Moorman *et al*, 2003; Steingart *et al*, 2003; Chien *et al*, 2005), but recall of prescription drugs varies according to the type of drug, the frequency of use, time since medication use, and education level (Saunders *et al*, 1994; West *et al*, 1995). Compared to pharmacy data, antidepressant use is substantially under-reported (Boudreau *et al*, 2004). A study comparing self-reported use of antihypertensives, statins, and antidepressants with pharmacy data found higher agreement for antihypertensives and statins than for antidepressant medications. Antidepressant use was recalled only 62% of the time within 6 months prior to reference date, compared to 94% for antihypertensive use (Boudreau *et al*, 2004). In addition, recall of names and dose of medication is relatively low (West *et al*, 1995) and the percentage missing timing of use is relatively high (Boudreau *et al*, 2004).

One strength of this study is the use of the GHC pharmacy records. Advantages of using administrative pharmacy records include having information available for all subjects (not only respondents), no under-reporting of use, no recall bias, and very complete information on drug name, dose, and timing of use. As most studies that use pharmacy databases to assess medication exposure have limited information on potential confounding factors (Wang *et al*, 2001; Sharpe *et al*, 2002; Gonzalez-Perez and Garcia Rodriguez, 2005; Haque *et al*, 2005), another important strength of the current study is the availability of breast cancer risk factor data.

One potential weakness of this study is the possibility that some women may have been prescribed antidepressants that were not included in the GHC pharmacy database. Prescriptions that were filled prior to the initiation of the pharmacy database in 1977, prior to enrollment in GHC, or filled out of the GHC system were not captured in the pharmacy databases. However, the number of antidepressants prescriptions filled outside the GHC system is likely to be small because antidepressant prescriptions were available free or with a small copayment for many GHC members. Further, others have reported that over 95% of prescriptions for antidepressants among GHC members are filled at a GHC pharmacy (Saunders *et al*, 1994). While we could not identify antidepressant use prior to GHC membership, we conducted subset analyses among women with at least 10 years of pharmacy data to ensure more complete history of antidepressant use. Over 70% of the subjects in this study were enrolled for more than 10 years as of their reference date and the conclusions based on this restricted sample were unchanged.

Another potential limitation of this study is a relatively short follow-up time for paroxetine exposure. Paroxetine was on the market in the United States in 1993, but because it was not a preferred drug in the GHC formulary, it was not used often within GHC until 1997. As a result, fewer subjects in this study population had ever used paroxetine and the duration of use was shorter than what would be expected in the general population during the same time period.

Overall, the results from this and other epidemiologic studies do not provide evidence for an increased risk of breast cancer associated with the use of antidepressants overall, by class of drug, or for individual antidepressants. Although a few studies have reported elevated risks in select analyses, the findings have not been consistent by class of drug, type of individual drug, or timing of use. The lack of consistency across studies suggests that any observed elevated risks could be due to chance. If there were a causal relationship, breast cancer risk would likely increase with longer duration of exposure. A few studies have reported elevated risks in longer-term users of some agents (Cotterchio *et al*, 2000; Moorman *et al*, 2003), but the class has differed. Overall, risk of breast cancer has not varied in any systematic pattern by timing of antidepressant use. As SSRIs have only been available in the relatively recent past, we cannot rule out the possibility of an effect with longer duration or longer time since first use, but the accumulating evidence does not suggest an association.

## Figures and Tables

**Table 1:** Characteristics of women with invasive breast cancer and controls

	**Cases (*N*=2449)**		**Controls (*N*=14396)**	
	***n***	**%**	***n***	**%**
*Age at diagnosis/reference date*				
30–39	92	3.8	507	3.5
40–49	461	18.8	2878	20.0
50–59	611	25.0	3554	24.7
60–69	618	25.2	3646	25.3
70+	667	27.2	3811	26.5
				
*Length of enrollment*				
4–9	642	26.2	3781	26.3
10–19	1007	41.1	5900	41.0
20+ years	800	32.7	4715	32.8
				
*First degree family history of breast cancer*				
Yes	452	20.6	1697	13.5
No	1746	79.4	10833	86.5
Missing	251	(10.3)	1866	(13.0)
				
*Age at menarche*				
⩽10	141	6.0	740	5.6
11–14	1936	83.0	10879	82.5
15+	256	11.0	1566	11.9
Missing	116	(4.7)	1211	(8.4)
				
*Parous*				
Yes	1910	85.9	11037	87.6
No	314	14.1	1568	12.4
Missing	225	(9.2)	1791	(12.4)
				
*Age at first birth (among parous women)*				
<20	374	19.6	2174	19.7
20–24	816	42.7	4804	43.5
25–29	443	23.2	2709	24.6
30–34	218	11.4	970	8.8
35+	58	3.0	377	3.4
Missing	1	(0.05)	4	(0.04)
				
*Use of birth control pills*				
Yes	1131	53.8	6301	52.0
No	973	49.7	5821	48.0
Missing	345	(14.1)	2274	(15.8)
				
*Ever use of HRT*				
Yes	1095	50.3	5352	43.8
No	1083	49.7	6858	56.3
Missing	271	(11.1)	2186	(15.2)
				
*Duration of use of HRT*				
⩾5 years	632	29.0	2901	23.8
< 5 years	443	20.3	2348	19.2
Duration unknown	20	0.9	103	0.8
None	1083	49.7	6858	56.2
Missing	271	(11.1)	2186	(15.2)
				
*Body mass index*				
Normal (<25)	1005	46.0	6101	49.7
Overweight (25–29.9)	665	30.4	3536	28.8
Obese (30+)	516	23.6	2631	21.5
Missing	263	(10.7)	2128	(14.8)
				
*Race*				
White	1859	90.8	9768	89.1
Asian	87	4.3	568	5.2
Black	57	2.8	351	3.2
Multi/other	44	2.2	275	2.5
Missing	402	(16.4)	3434	(23.9)
				
*Education*				
Less than high school	143	6.7	792	7.2
High school graduate	499	23.3	2627	23.8
Some post secondary	736	34.3	3851	34.9
College graduate	350	16.3	1687	15.3
Advanced degree/some graduate school	418	19.5	2094	19.0
Missing	303	(12.4)	3345	(23.2)
				
*Previous biopsy*				
Yes	574	25.9	2156	17.2
No	1640	74.1	10406	82.8
Missing	235	(9.6)	1834	(12.7)
				
*At least 1 screening mammogram within 2 years prior to reference date*				
Yes	1728	70.6	8370	58.1
No	721	29.4	6026	41.9

**Table 2:** Characteristics of antidepressant users and nonusers among controls

	**Antidepressant users (*N*=3426)**		**Nonusers (*N*=10970)**	
	***n***	**%**	***n***	**%**
*Age at reference date*				
30–39	70	2.0	437	4.0
40–49	635	18.5	2243	20.5
50–59	910	26.6	2644	24.1
60–69	845	24.7	2801	25.5
70+	966	28.2	2845	25.9
				
*Length of enrollment*				
4–9	745	21.8	3036	27.7
10–19	1422	41.5	4478	40.8
20+ years	1259	36.8	3456	31.5
				
*First degree family history of breast cancer*				
Yes	487	15.6	1210	12.9
No	2638	84.4	8195	87.1
				
*Age at menarche*				
⩽10	218	6.8	522	5.2
11–14	2653	82.2	8226	82.6
15+	358	11.1	1208	12.1
				
*Parous*				
Yes	2759	87.6	8278	87.5
No	389	12.4	1179	12.5
				
*Age at first birth*				
<20	684	24.8	1490	18.0
20–24	1229	44.6	3575	43.2
25–29	581	21.0	2128	25.7
30–34	189	6.9	781	9.4
35+	76	2.8	301	3.6
				
*Ever use of HRT*				
Yes	1638	53.8	3714	40.5
No	1405	46.2	5453	59.5
				
*Duration of use of HRT*				
⩾5 years	923	30.8	1978	21.7
<5 years	673	22.4	1675	18.4
None	1405	46.8	5453	59.9
				
*Body mass index*				
Normal (<25)	1359	44.2	4742	51.6
Overweight (25–29.9)	855	27.8	2681	29.2
Obese (30+)	861	28.0	1770	19.3
				
*Race*				
White	2502	91.3	7266	88.4
Asian	74	2.7	494	6.0
Black	84	3.1	267	3.3
Multi/other	81	3.0	194	2.4
				
*Education*				
Less than high school	221	8.0	571	6.9
High school graduate	683	24.6	1944	23.5
Some post secondary	1004	36.1	2847	34.4
College graduate	398	14.3	1289	15.6
Advanced degree/some graduate school	473	17.0	1621	19.6
				
*Previous biopsy*				
Yes	592	18.9	1564	16.6
No	2547	81.1	7859	83.4
				
*At least 1 screening mammogram within 2 years prior to reference date*				
Yes	2175	63.5	6195	56.5
No	1251	36.5	4775	43.5

**Table 3:** Antidepressant use among women with invasive breast cancer and controls

	**Cases (*N*=2449)**	**Controls (*N*=14396)**	**Adjusted for matching factors^a^ (*N*=16845)**		**Adjusted for potential confounding factors^b^ (*N*=13872)**	
**Exposure variables**	***n***	***n***	**OR**	**95% CI**	**OR**	**95% CI**
No antidepressants	1822	10970	1.00		1.00	
						
*Antidepressants*						
Any use	627	3426	1.11	1.00–1.22	1.04	0.94–1.16
2–10 prescriptions	276	1503	1.11	0.97–1.27	1.04	0.90–1.21
11–20 prescriptions	120	627	1.16	0.95–1.42	1.10	0.89–1.38
21–50 prescriptions	126	760	1.01	0.83–1.22	0.94	0.76–1.16
51+ prescriptions	105	536	1.19	0.96–1.48	1.12	0.89–1.41
						
*Tricyclics*						
Any use	527	2850	1.12	1.00–1.24	1.06	0.94–1.19
2–10 prescriptions	273	1484	1.11	0.97–1.28	1.05	0.90–1.22
11–20 prescriptions	94	490	1.16	0.92–1.45	1.13	0.89–1.44
21–50 prescriptions	94	557	1.02	0.82–1.28	0.96	0.76–1.23
51+ prescriptions	66	319	1.25	0.96–1.64	1.16	0.86–1.55
						
*SSRIs*						
Any use	154	911	1.04	0.86–1.24	0.98	0.80–1.18
2–10 prescriptions	78	461	1.03	0.81–1.32	0.97	0.74–1.26
11–20 prescriptions	35	203	1.06	0.73–1.52	0.90	0.60–1.33
21+ prescriptions	41	247	1.02	0.73–1.44	1.04	0.73–1.48
						
*Atypical*						
Any use	150	876	1.04	0.87–1.25	0.95	0.78–1.16
2–10 prescriptions	90	535	1.02	0.81–1.29	0.93	0.72–1.16
11–20 prescriptions	23	135	1.04	0.99–1.92	1.10	0.68–1.73
21–50 prescriptions	24	142	1.03	0.66–1.59	0.82	0.50–1.34
51+ prescriptions	13	64	1.25	0.68–2.27	1.13	0.60–2.13

a Adjusted for age, length of enrollment, and calendar year.

b Adjusted for age, length of enrollment, calendar year, family history of breast cancer, parity/age at first birth, duration of HRT use, body mass index, and history of screening mammogram in 2 years prior to reference date.

**Table 4:** Use of specific antidepressant medications among women with invasive breast cancer and controls

	**Cases**	**Controls**	**Adjusted for matching factors^a^**		**Adjusted for potential confounding factors^b^**	
**Exposure variable**	***n***	***n***	**OR**	**95% CI**	**OR**	**95% CI**
No antidepressants	1822	10970	1.00		1.00	
						
*Tricyclics*						
Amitriptyline						
Any use	250	1191	1.27	1.10–1.47	1.21	1.03–1.41
2–10 prescriptions	151	756	1.20	1.00–1.44	1.17	0.97–1.43
11+ prescriptions	99	435	1.38	1.10–1.72	1.26	0.99–1.60
						
Doxepin						
Any use	186	1101	1.01	0.86–1.19	0.95	0.79–1.13
2–10 prescriptions	120	710	1.01	0.83–1.23	0.94	0.75–1.17
11+ prescriptions	66	391	1.01	0.78–1.32	0.96	0.72–1.27
						
Imipramine						
Any use	127	683	1.13	0.93–1.37	1.04	0.84–1.29
2–10 prescriptions	79	444	1.08	0.84–1.38	0.98	0.76–1.28
11+ prescriptions	48	239	1.22	0.89–1.67	1.15	0.82–1.61
						
*SSRIs*						
Fluoxetine						
Any use	114	611	1.07	0.86–1.34	1.00	0.80–1.25
2–10 prescriptions	59	364	0.99	0.75–1.31	0.94	0.69–1.27
11+ prescriptions	55	297	1.14	0.85–1.53	1.07	0.78–1.46
						
Paroxetine						
Any use	41	241	1.05	0.75–1.48	1.00	0.70–1.41
2–10 prescriptions	36	178	1.25	0.87–1.81	1.15	0.79–1.69
11+ prescriptions	5	63	0.49	0.20–1.23	0.52	0.21–1.31
						
Sertraline						
Any use	40	215	1.15	0.82–1.62	1.16	0.81–1.66
2–10 prescriptions	24	129	1.15	0.74–1.78	1.18	0.75–1.86
11+ prescriptions	16	86	1.15	0.67–1.98	1.12	0.64–1.98
						
*Atypical*						
Trazodone						
Any use	141	802	1.07	0.89–1.29	0.96	0.78–1.18
2–10 prescriptions	82	502	0.99	0.78–1.26	0.88	0.68–1.15
11+ prescriptions	59	300	1.20	0.90–1.59	1.09	0.80–1.49

a Adjusted for age, length of enrollment, and calendar year.

b Adjusted for age, length of enrollment, calendar year, family history of breast cancer, parity/age at first birth, duration of HRT use, body mass index, history of screening mammogram in 2 years prior to reference date.

**Table 5:** Antidepressant use among women with invasive breast cancer, by ER status, and controls

	**ER+ cases**	**ER– cases**	**Controls**	**ER+ compared to controls^a^**		**ER− compared to controls^a^**	
**Exposure variables**	***n***	***n***	***n***	**OR**	**95% CI**	**OR**	**95% CI**
No antidepressants	1307	325	10970	1.00		1.00	
							
*Antidepressant*							
Any use	457	106	3426	1.05	0.93–1.19	1.01	0.79–1.30
2–10 prescriptions	207	45	1503	1.09	0.92–1.29	0.93	0.65–1.33
11–20 prescriptions	95	16	627	1.19	0.94–1.51	0.87	0.50–1.50
21–50 prescriptions	84	25	760	0.85	0.67–1.10	1.05	0.66–1.65
51+ prescriptions	71	20	536	1.04	0.79–1.36	1.39	0.86–2.26
							
*Tricyclic*							
Any use	386	84	2850	1.07	0.94–1.21	0.95	0.72–1.24
2–10 prescriptions	200	48	1484	0.97	0.68–1.38	1.06	0.90–1.26
11–20 prescriptions	74	12	490	0.92	0.51–1.66	1.19	0.92–1.55
21–50 prescriptions	67	10	557	0.55	0.27–1.13	0.94	0.72–1.25
51+ prescriptions	45	14	319	1.63	0.91–2.90	1.09	0.77–1.60
							
*SSRI*							
Any use	108	28	911	0.93	0.74–1.16	1.05	0.69–1.60
2–10 prescriptions	55	13	461	0.92	0.68–1.24	1.05	0.59–1.87
11–20 prescriptions	26	7	203	0.95	0.61–1.47	0.87	0.35–2.15
21+ prescriptions	27	8	247	0.93	0.62–1.41	1.21	0.58–2.51
							
*Atypical*							
Any use	108	31	876	0.92	0.73–1.16	1.15	0.76–1.74
2–10 prescriptions	66	19	535	0.93	0.70–1.23	1.11	0.66–1.86
11–20 prescriptions	17	6	135	1.06	0.62–1.80	1.76	0.76–4.07
21+ prescriptions	25	6	206	0.82	0.52–1.31	0.89	0.36–2.20
							
*Amitriptyline*							
Any use	186	38	1191	1.22	1.02–1.45	1.09	0.75–1.58
2–10 prescriptions	113	23	756	1.19	0.95–1.48	1.05	0.66–1.67
11+ prescriptions	73	15	435	1.27	0.96–1.66	1.16	0.65–2.06

a Adjusted for age, length of enrollment, calendar year, family history of breast cancer, parity/age at first birth, duration of HRT use, body mass index, and history of screening mammogram in 2 years prior to reference date.
